# Structural and Biochemical Characterization of BdsA from *Bacillus subtilis* WU-S2B, a Key Enzyme in the “4S” Desulfurization Pathway

**DOI:** 10.3389/fmicb.2018.00231

**Published:** 2018-02-15

**Authors:** Tiantian Su, Jing Su, Shiheng Liu, Conggang Zhang, Jing He, Yan Huang, Sujuan Xu, Lichuan Gu

**Affiliations:** ^1^State Key Laboratory of Microbial Technology, School of Life Sciences, Shandong University, Jinan, China; ^2^Faculty of Light Industry, Province Key Laboratory of Microbial Engineering, Qilu University of Technology (Shandong Academy of Sciences), Jinan, China

**Keywords:** FMN-binding, dibenzothiophene, desulfurization, monooxygenase, “4S” pathway

## Abstract

Dibenzothiophene (DBT) and their derivatives, accounting for the major part of the sulfur components in crude oil, make one of the most significant pollution sources. The DBT sulfone monooxygenase BdsA, one of the key enzymes in the “4S” desulfurization pathway, catalyzes the oxidation of DBT sulfone to 2′-hydroxybiphenyl 2-sulfonic acid (HBPSi). Here, we determined the crystal structure of BdsA from *Bacillus subtilis* WU-S2B, at the resolution of 2.2 Å, and the structure of the BdsA-FMN complex at 2.4 Å. BdsA and the BdsA-FMN complex exist as tetramers. DBT sulfone was placed into the active site by molecular docking. Seven residues (Phe12, His20, Phe56, Phe246, Val248, His316, and Val372) are found to be involved in the binding of DBT sulfone. The importance of these residues is supported by the study of the catalytic activity of the active site variants. Structural analysis and enzyme activity assay confirmed the importance of the right position and orientation of FMN and DBT sulfone, as well as the involvement of Ser139 as a nucleophile in catalysis. This work combined with our previous structure of DszC provides a systematic structural basis for the development of engineered desulfurization enzymes with higher efficiency and stability.

## Introduction

The burning of fossil fuels causes worldwide environmental pollution because of the sulfur compounds present in fossil fuels. Hydrodesulfurization (HDS) can remove sulfur from petroleum, but heterocyclic sulfur compounds are difficult to remove completely. Thus, reducing the presence of heterocyclic sulfur compounds, such as dibenzothiophene (DBT) and alkylated DBTs, has become increasingly important. Physical and chemical methods of desulfurization are not economically viable and may cause additional environmental pollution. Therefore, biological desulfurization (BDS) for the treatment of recalcitrant organic sulfur compounds has gained attention (Monticello et al., 1985; Gray et al., 2003).

**FIGURE 1:**
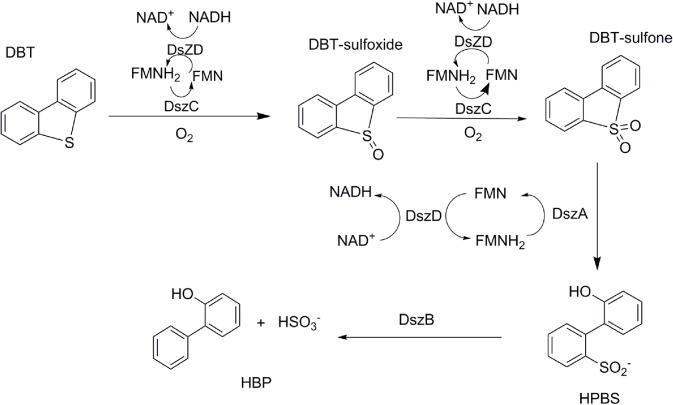
Primary four-step 4S biodesulfurization pathway as adapted from Oldfield et al. (1997).

Numerous efforts have been focused on investigating biodesulfurization systems, using DBT or its alkylated derivatives as model compounds. The pathway specifically cleaving the C-S bond during metabolic desulfurization has been termed the “4S” pathway (**Figure 1**). The genes, involved in the metabolic desulfurization of the “4S” pathway (*dszABCD*), were first reported in *Rhodococcus* sp. IGTS8 (Denome et al., 1993; Kayser et al., 1993). In the initial step of the desulfurization process, DBT is oxidized into DBT sulfone by DBT monooxygenase DszC. Then, DBT sulfone monooxygenase DszA catalyzes the oxidation of DBT sulfone into 2′-hydroxybiphenyl 2-sulfonic acid (HBPSi). Finally, HBPSi is desulfurized by the HBPSi desulfinase DszB to 2-hydroxybiphenyl (2-HBP) and sulfite (Denome et al., 1994; Gray et al., 1998). Some thermophilic desulfurizing bacteria, such as *Bacillus subtilis* WU-S2B, *Paenibacillus,* and *Pseudomonas*, can grow in the presence of DBT, utilizing DBT as their sole source of sulfur (Konishi et al., 1997; Ishii et al., 2000a; Kahnert and Kertesz, 2000). The DBT desulfurization enzymes, TszABCD, have been isolated from *Paenibacillus* (Ishii et al., 2000b). TszABCD have higher heat stability than DszABCD, but the enzymatic activities of TszABCD are lower than those of DszABCD. The corresponding genes from *Bacillus subtilis* WU-S2B have been identified and the enzymes (BdsABCD) characterized (Kirimura et al., 2001). Additionally, recombinant *Escherichia coli* cells harboring the *bdsABCD* genes exhibit DBT desulfurizing activity at 50°C, and purified BdsA and BdsB have higher enzymatic activities than those of DszA and DszB (Kirimura et al., 2004; Ohshiro et al., 2005). The DNA sequences of BdsABC share high identities (52–73%) with those of DszABC and TdsABC. Multiple conserved proteins motifs have also been identified (Kilbane and Robbins, 2007). These most highly conserved regions could be used to construct improved universal primers for the PCR detection/cloning of bdsA/dszA genes in uncharacterized biodesulfurization-competent microbial cultures, which may help identify possible biodesulfurization genes in genomic data and provide an aid to the annotation of genes in the desulfurization pathway.

The reactions, catalyzed by DBT desulfurization enzymes, have been characterized (Gallagher et al., 1993; Yan et al., 2000; Gray et al., 2003; Kilane, 2006). DszA and DszC belong to Group C of the family of flavin-dependent monooxygenases which contain a TIM-barrel fold and receive reduced flavin from a NAD(P)H-dependent reductase (Montersino et al., 2011; Huijbers et al., 2014). The oxidoreductase DszD supplies the NADH necessary for the activity of monooxygenases DszA and DszC. DszB is a hydrolase that participates in the last desulfurization step and hydrolyzes 2′-hydroxybiphenyl-2-sulfinic acid into 2-hydroxybiphenyl and sulfite. During preliminary characterization research of these enzymes, the results showed that DszB is the rate-limiting enzyme (Abin-Fuentes et al., 2014; Kilbane, 2016). Biological desulfurization can make greater contributions in future, but there exist some issues regarding the catalytic mechanism of key enzymes in the “4S” pathway that need to be resolved.

The structures of these key enzymes, involved in the “4S” pathway, have been studied extensively. The structure of DszB was first reported in 2006, and the reaction mechanism was further illustrated (Lee et al., 2006). The crystal structures of DszC and its complex with FMN have also been reported (Liu et al., 2014; Zhang et al., 2014; Guan et al., 2015), and the homology TdsC has also been reported (Hino et al., 2017). Recently, a liquid chromatography–mass spectrometry analysis showed that the C2 hydroperoxide of the DBT sulfone reacts with reduced flavin to form a flavin-N5-oxide intermediate in DszA, which is involved in subsequent protonation (Adak and Begley, 2016). However, without the structure of BdsA, it is unknown how this process happens in the active site of BdsA and how the protein enzyme facilitates the reaction.

To further understand the catalytic mechanism of DBT sulfone monooxygenation, we determined the crystal structure of BdsA from *Bacillus subtilis* WU-S2B at the resolution of 2.2 Å, and the structure of the BdsA-FMN complex at 2.4 Å. Site-directed mutagenesis showed that mutations in the residues involved in catalysis or in flavin substrate-binding result in a significant loss of enzymatic activity, which provides valuable clues for elucidating the catalytic mechanism of DBT sulfone monooxygenase.

## Materials and Methods

### Cloning, Protein Expression, and Purification

The *bdsA* gene, from *Bacillus subtilis* WU-S2B, was synthesized and used as the template for PCR amplification. The amplified *bdsA* was inserted into the NdeI and XhoI sites of a pET-15b vector (Novagen) in frame with an N-terminal His-tag; the resulting plasmid was transformed into *E. coli* BL21 (DE3) cells for BdsA overexpression.

*Escherichia coli* BL21 (DE3) cells, harboring the *bdsA* overexpression plasmid, were grown in Luria-Bertani (LB) medium supplemented with 100 μg/mL ampicillin. The cells were induced by 0.12 mM isopropyl β-D-1-thiogalactopyranoside (IPTG) when the OD_600_ reached 0.9, and were incubated overnight at 22°C. Cells were subsequently harvested by centrifugation (8400 *g* for 15 min) and lysed by ultrasonication in lysis buffer [25 mM Tris-HCl, pH 8.0; 200 mM NaCl; 0.4 mM phenylmethylsulfonyl fluoride (PMSF)]. After centrifugation at 28,000 *g* for 45 min at 4°C, the supernatant was applied to a Ni-NTA affinity column (GE Healthcare) equilibrated in the lysis buffer. The 6× His-tagged BdsA was eluted with elution buffer (25 mM Tris-HCl, pH 8.0; 100 mM NaCl; 250 mM imidazole), followed by further purification by anion exchange on a 16/10 HR Source-15Q column (GE Healthcare) with gradient buffer (Buffer A: 25 mM Tris-HCl, pH 8.0; Buffer B : 25 mM Tris-HCl, pH 8.0, 1 M NaCl), and then by size-exclusion chromatography using a gel-filtration column (Superdex 200 10/300 GL, GE Healthcare) in 10 mM Tris-HCl pH 8.0, 100 mM NaCl. Purified BdsA was analyzed by SDS-PAGE followed by Coomassie brilliant blue staining (R250). The concentrations of purified proteins were determined using Nanodrop (A-280 nm) (Thermo Scientific).

### Site-Directed Mutagenesis of BdsA

Site-directed mutagenesis of BdsA was conducted by two-step PCR using the wild-type pET-15b/*bdsA* plasmid as template. The mutant clones were confirmed by DNA sequencing (Invitrogen). The expression and purification processes were the same as that for the wild-type BdsA protein.

### Crystallization and Data Collection

For crystallization, the purified BdsA proteins were concentrated to 10–20 mg/mL measured by Nanodrop (A-280nM). Crystals were first screened by sitting drop vapor diffusion using crystallization screen kits (Hampton Research) at 20°C. After optimization, native crystals were obtained from the hanging drops by mixing equal volumes of the protein solution and reservoir solution [0.1 M sodium citrate (pH 5.5) and 35% PEG 200]. To obtain crystals of the BdsA-FMN complex, native crystals were soaked in the solution, containing 0.1 M sodium citrate (pH 5.5), 35% PEG 200 and 1 mM FMN, for 30 min. We also tried to obtain the crystals of the BdsA-FMN-substrate complex by the same means. Unfortunately, the efforts failed.

For data collection, crystals were flash-frozen in liquid nitrogen, with 15–20% (v/v) ethylene glycol used as cryoprotectant. The X-ray diffraction data sets were collected at 100K on a beam line BL17U at the Shanghai Synchrotron Radiation Facility (Shanghai, China) equipped with an ADSC Q315r CCD-detector.

### Structure Determination and Refinement

The X-ray diffraction data were integrated and scaled using the HKL-2000 program suite (Otwinowski and Minor, 1997). The native BdsA structure was resolved by molecular replacement using Phaser from the CCP4 suit of programs (Winn et al., 2011) with LadA (PDB entry 3B9N) as the search model. Refinement was performed using the PHENIX crystallography suite (Adams et al., 2002) and the COOT interactive model-building program (Emsley and Cowtan, 2004). The final R-values were R_work_ = 17.47% and R_free_ = 21.30% based on a subset of 5% of the reflections. The cofactor FMN was added to the complex model, based on the F*o*–F*c* density map of the ligand structure, and refinement was conducted in the same manner as that for apo-BdsA. The final model had a R_work_ = 18.11% and R_free_ = 22.11% based on a subset of 5% of the reflections.

Diffraction data collection and refinement statistics are listed in **Table 1**. The final models were checked and validated using PROCHECK (Laskowski et al., 1993), QMEAN (Benkert et al., 2008), and ProQ (Cristobal et al., 2001) model quality assessment tools, which indicated that the models were of good quality. Structure graphics were illustrated with the PyMOL molecular visualization system (Lill and Danielson, 2011). The atomic coordinates and structure factors of BdsA and the BdsA-FMN complex were deposited in the Protein Data Bank with accession codes 5XKC and 5XKD, respectively.

**Table 1:** X-ray data collection and refinement statistics.

Data collection	Apo	FMN-bound
Space group Unit-cell parameters	P21212	P21212
a, b, c (Å)	132.494, 174.574, 85.345	131.616, 175.890, 84.934
α, β, γ (°)	90.00, 90.00, 90.00	90.00, 90.00, 90.00
Wavelength (Å)	0.9870	0.9870
Resolution (Å)	50.00-2.20 (2.28-2.20)	50-2.40 (2.49-2.40)
Unique reflections	98498 (9746)	78295 (7724)
Completeness (%)	99.9 (100)	99.9 (99.9)
Redundancy	6.7 (6.5)	6.8 (6.6)
*I/σ(I)*	33.89 (4.72)	29.07 (4.21)
*R_merge_* (%)^a^	9.4 (57.0)	10.5 (56.9)
**Refinement**		
*R*_working_ (%)	17.47	18.11
*R*_free_ (%)	21.30	22.11
No. of		
Protein residues	1784 (homotetramer)	1784 (homotetramer)
FMN		4
solvent	446	250
R.m.s.d. from ideal geometry		
Bond length (Å)	0.007	0.010
Bond angles (°)	1.030	1.440
Wilson *B*-value (Å^2^)	33.48	36.17
Average *B*-factors (Å^2^)		
Protein	41.27	48.79
FMN		47.44
Solvent	40.10	45.04
Ramachandran plot (%)		
Favored	96.85	97.40
Allowed	2.98	2.54
Outliers	0.17	0.06

^a^*R_*merge*_* = Σ_*hkl*_Σ_*i*_|*I_*i*_(hkl)*-<*I(hkl)*>|/Σ_*hkl*_Σ_*i*_I_*i*_(hkl). where <*I(hkl)*> *is the mean intensity of multiply recorded reflections.*

*The coordinates and structure factors have been deposited in the Protein Data Bank with the accession code 5XKC (apo-BdsA) and 5XKD (FMN-bound).*

### Calculations of Enzyme-Substrate Molecular Docking

AutoDock 4.2 was used for the molecular docking calculations (Morris et al., 2009). The substrate coordinates were designed using the Dundee PROGRG server (Schuttelkopf and van Aalten, 2004). The DBT sulfone docking was based on the BdsA-FMN complex structure. The ligands and BdsA were prepared using AutoDockTools, and the BdsA was designed to be rigid. Polar hydrogens and Kollman United Atom charges were added to the enzyme; Gasteiger charges were assigned; and non-polar hydrogen atoms were merged for the ligands. Docking calculations were performed using the default settings for the genetic algorithm parameters with 25,000,000 energy evaluations per run. A composite file of all possible conformers was analyzed by AutoDock Tools. The chemical reasonableness of best results was evaluated by examining the interactions between the BdsA and best-docked conformer.

### Enzymatic Activity Assays

The activity of BdsA was determined by measuring the amount of HBPSi using high-performance liquid chromatography (HPLC) according to a method described previously (Ohshiro et al., 1997, 1999). The enzyme reaction system for measuring the activity of BdsA contained 100 mM potassium phosphate buffer (pH 7.0), 0.25 mM DBT sulfone, 6 mM NADH, 10 μM FMN, 20 nM Fre (flavin reductase from *E. coli* O157), and 2 μM BdsA, in a total volume of 1 mL. The reaction was performed with rotary shaking (2000 rpm) at 35°C for 20 min, and stopped by the addition of 100 μL of 12 M HCl. The mixture was extracted with ethyl acetate, centrifuged at 15,000 *g* for 5 min, and then the supernatant was injected into an HPLC system using 20 mM KH_2_PO_4_ (pH 2.5) and methanol as the mobile phase, at a ratio of 2:3.

The reaction mixture was loaded onto a C18 reversed-phase column (Venusil XBP-C18, Agela Technologies) attached to an HPLC system (LC-10AT pump, SPD-10A UV/VIS detector). An increase in absorbance at 280 nm, which indicates the amount of HBPSi, was observed. The enzymatic assays for all mutants were performed in the same manner as that for the wild-type BdsA protein.

## Results

### Overall Structure of apo-BdsA

**FIGURE 2:**
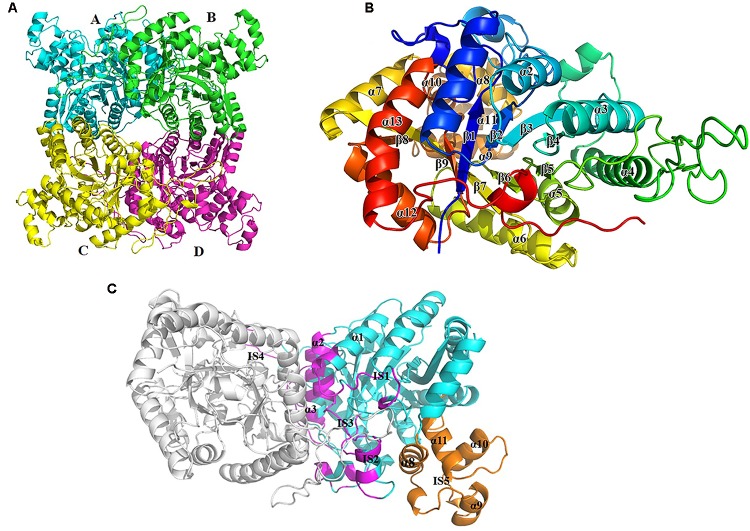
Overall structure of BdsA. **(A)** The four BdsA monomers (A, B, C, and D) forming two homodimers are designated with cartoon in different colors. **(B)** The secondary structures (13 α-helices and 9 β-strands) of BdsA monomer are depicted in rainbow colored cartoon. **(C)** Structure of BdsA dimer. The dimer is shown in cartoon model. Molecule A is in gray color and molecule B is in blue color. The dimer interface consists of α1, IS1, IS2, α2, α3, IS3, and IS4. IS5 separated from the protein core is located between α7 and β8, which contains four small helices (α8, α9, α10, and α11).

The crystal structure of apo-BdsA (unbound structure) from *Bacillus subtilis* belongs to the P21212 space group with the cell dimension a = 132.494 Å, b = 174.574 Å, and c = 85.345 Å. The asymmetric unit contains four peptide chains, designated A, B, C, and D, which form two homodimers, AB and CD (**Figure 2A**). Each BdsA monomer is composed of 453 amino acids. In the structure of BdsA, the electron densities for the N-terminus to Gln4 and Ser451 to the C-terminus are not visible, likely because these regions are flexible and disordered. In the final model of apo-BdsA, more than 96.85% of the residues were located in the favored regions of the Ramachandran plot and only 2.98% in the generous and allowed regions.

The BdsA monomer contains nine β-strands (β1–β9) and 13 α-helices (α1–α13), which fold into a triosephosphate isomerase (TIM)-barrel with five extended insertion regions (**Figure 2B**). These extended insertion regions are located in the connecting regions between β-strands and α-helices, designated as insertion segments IS1 to IS5 (**Figure 2C**). IS1 is located between β1 and α1, IS2 between β2 and α2, IS3 between β3 and α3, and IS4 between α4 and β5. According to the structure, IS1 to IS4 play important roles in the formation of the BdsA homodimer (**Figure 2C**). IS5 is located between α7 and β8, which contain four small helices (α8, α9, α10, and α11). IS5, the largest insertion region, is separated from the protein core and forms a deep groove with the protein core (**Figures 2B,C**). The deep groove is important in FMN-binding and substrate catalysis. IS5 forms the entrance for FMN and the substrate.

### Tetramer Structure of BdsA

The crystal structure of BdsA shows that there are four molecules in an asymmetric unit (**Figure 2A**). To determine its oligomeric state in solution, we performed gel-filtration experiments using a Superdex-200 column (GE Healthcare) running in 10 mM Tris-HCl (pH 8.0) and 100 mM NaCl. The elution volume of BdsA indicated a molecular mass of ∼220 kDa, indicating that BdsA exists as a tetramer in the solution.

Structure comparison showed that the two homodimers, AB and CD, have a high structural similarity. The root-mean-square deviation (RMSD) value for C_α_ is 0.165 Å. As predicted by PISA (Krissinel and Henrick, 2005), the AB dimer has a surface area of approximately 3,177 Å^2^, and CD has a surface area of approximately 3,217 Å^2^. The dimer interface is extensive and consists predominantly of hydrophobic residues, which are derived from α1, IS1, IS2, α2, α3, IS3, and IS4 (**Figure 2C**).

### Structure of the BdsA–FMN Complex

**FIGURE 3:**
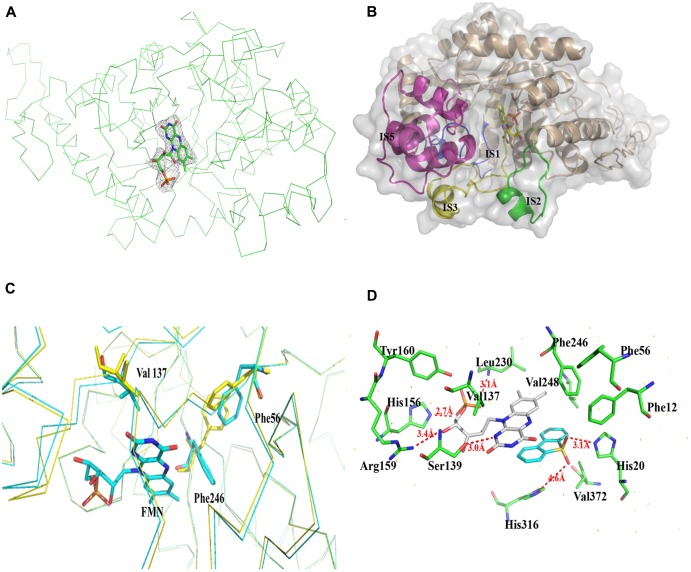
Structure of BdsA-FMN complex. **(A)** The electron density map (contoured at 2.0σ) reveals the existence of FMN within BdsA active pocket. **(B)** The pocket constituting of IS1, IS2, IS3, and IS5 forms the active site of FMN and substrate binding. **(C)** Structure comparison of the apo and FMN bound BdsA. Yellow ribbon represents the apo-BdsA structure and cyan ribbon stands for the FMN bound structure. The FMN is depicted in color sticks. Distinct structural rearrangements occur at residues Phe56, Val137, and Phe246. **(D)** The docking result of the BdsA-FMN-DBT sulfone complex. Residues constituting the binding pocket are shown as green sticks. The cofactor FMN is shown as gray sticks. DBT sulfone is shown as cyan sticks. V137, S139, H156, R159, Y160, and L230 play significant roles in FMN binding. F12, F56, F246, V248, H316, and V372 play directional roles in substrate binding.

The structure of the tetrameric BdsA–FMN complex contains four FMN molecules, with each subunit binding one FMN (**Figure 3A**). The TIM-barrel structure confirms that BdsA belongs to the group C flavin-dependent monooxygenases. The binding pocket is mainly formed by IS1, IS2, IS3, and IS5 (**Figure 3B**). The overall C_α_ RMSD between apo-BdsA and the BdsA-FMN complex is only 0.240 Å. The most substantial structural discriminations were observed at the side chains of residues Phe56, Val137, and Phe246 (**Figure 3C**), which may facilitate the binding of FMN.

The binding of FMN to BdsA is maintained through hydrogen bonds and hydrophobic interactions. The flavin ring of FMN lies in the barrel, with its plane nearly parallel with the staves of the barrel, and its si-face exposed to the solvent. There are several hydrogen bonding interactions between BdsA and FMN: the NE2 atom of His 156 with the O3 atom of the flavin phosphate groups (2.7 Å); the OG atom of Ser139 with the N1 atom of the flavin ring (2.8 Å); backbone nitrogen of Leu230 with the phosphate group (3.1 Å); and the Arg159 with the O4 atom of the flavin ring (3.4 Å). Two residues, Tyr160 and Val137, form van der Waals contacts with FMN (**Figure 3D**).

**FIGURE 4:**
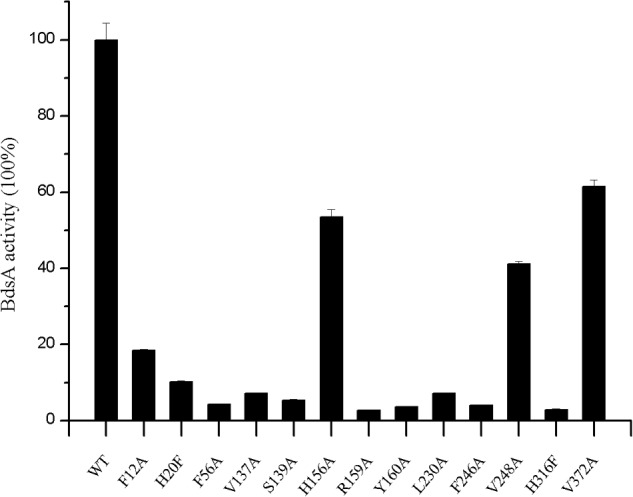
Effects of active site residue mutants on enzyme activity of BdsA. Activity is defined as the number of nmol of HBPSi produced min^-1^ nmol^-1^ in the presence of BdsA. Enzyme assays for all mutants were performed in the same way that the wild-type BdsA protein was tested. Error bars indicate the standard deviation of four repeats. The result figure was generated by origin pro 8.

Based on the structural analysis, six single-point mutations of BdsA were constructed: V137A, S139A, H156A, R159A, Y160A, and L230A. The secondary structures of mutant BdsA proteins were in good agreement with the wild type protein when examined by circular dichroism spectroscopy (**Supplementary Figure S1**). The results of enzymatic activity showed that mutant proteins V137A, S139A, R159A, Y160A, and L230A completely lost their activity (**Figure 4**). The activity of H156A was decreased by approximately 50% (**Figure 4**). We also showed the superposition of the wild type and variant amino acids, which indicated that all the point mutations significantly disrupt the interactions between the side chains of the active site residues and FMN (**Supplementary Figure S2**). These results indicate that these residues play significant roles in the binding of FMN, which was consistent with the results of the structural analysis.

### Substrate Docking and Analysis of Mutagenesis

The substrate DBT sulfone is the intermediate product of DBT desulfurization. It is difficult to crystallize the BdsA-FMN-substrate complex because DBT sulfone binds poorly to the oxidized form of the enzyme. To examine the binding and catalytic mechanism of DBT sulfone, we used flexible docking (Autodock 4.2) to build three-dimensional models of the BdsA-FMN-DBT sulfone complex. The docking result is illustrated in **Figure 3D**. As shown, the DBT sulfone substrate is located in the active site, on the si-face of the FMN isoalloxazine ring (**Supplementary Figure S3**). According to the results of the molecular docking analysis, seven residues (Phe12, His20, Phe56, Phe246, Val248, His316, and Val372) are involved in the binding of DBT sulfone (**Figure 3D**). The distance between flavin C4a and the carbon atom of the DBT sulfone attacked by the flavin hydroperoxide is 4.5 Å. The structure suggests that there is space between flavin C4a and the attacked atom of DBT sulfone for the formation of a (hydro) peroxyflavin intermediate (Li et al., 2008).

To illustrate the function of the substrate-binding residues, seven single-point mutants of BdsA were constructed: F12A, H20F, F56A, F246A, V248A, H316F, and V372A. The enzyme activities of these proteins were analyzed by measuring the formation of HBPSi on HPLC, and each assay was repeated four times (**Figure 4**). The results showed that mutant proteins H20F, F56A, F246A, and H316F lost more than 90% activity compared with wild-type BdsA. The activity of F12A was decreased by approximately 80%, and those of V248A and V372A were decreased by approximately 50%. These results were consistent with the results of the molecular docking calculations. Taken together, our results indicate that residues F12, H20, F56, F246, V248, H316, and V372 may play vital roles in substrate binding.

## Discussion

### The Overall Structure and Active Site of BdsA Are Highly Conserved

**FIGURE 5:**
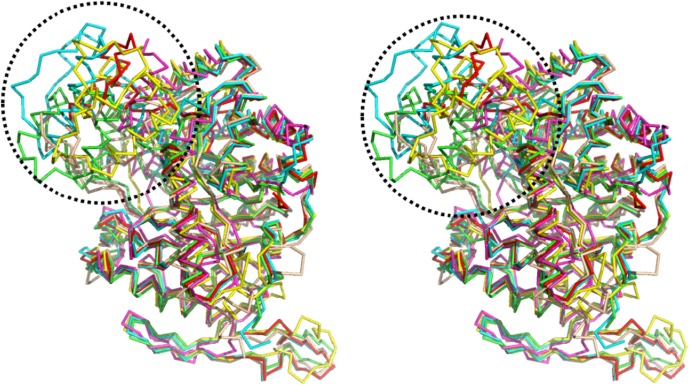
Structure comparison of BdsA with homology structures by the DALI program against Protein Data Bank. BdsA, Ytnj, Lada, Emoa, and Luciferase-like Monooxygenase are illustrated in green, cyan, red, yellow, and magenta, respectively. The most difference in the structure of BdsA range from Val304–Val375 (circled by black dashed line), including α8, α9, α10, and α11. It is the key component of the active pocket, which controls the size of the pocket accompanied with IS1, IS2, and IS3.

A structure-based alignment was conducted, using the DALI program (Holm and Rosenström, 2010), against the Protein Data Bank (PDB). The similarity results showed that the scaffold of BdsA is highly conserved among all the group C flavoprotein monooxygenase superfamily. The nitrilotriacetate monooxygenase (PDB: 3sdo Z-score 48.8) is most similar to BdsA, followed by Ytnj (PDB: 1ywl, Z-score 47.6), alkane monooxygenase (Lada, PDB: 3b9n, Z-score 46.9) (Li et al., 2008), ethylenediaminetetra acetic acid (EDTA) monooxygenase (Emoa, PDB: 5dqa, Z-score 44.2) (Macdonald et al., 2016), and luciferase-like monooxygenase (PDB: 3rao, Z-score 32.9). The C_α_ superposition of BdsA with Ytnj, Lada, Emoa, and luciferase-like monooxygenase yielded the RMSD values of 1.178, 1.322, 1.316, and 2.364 Å, respectively. The homologs fold as TIM-barrels and are highly conserved in structural folding. However, the structural identities of the insertion segment IS5 were very low (**Figure 5**). In BdsA, IS5 ranges from Val304 to Val375, including α8, α9, α10, and α11. It is the key component of the active pocket, which, along with IS1, IS2, and IS3, controls the size of the pocket. The volume of the active pocket of BdsA, and those of homologous proteins, are calculated using CASTp server (Dundas et al., 2006). The volume of the BdsA activity pocket is 5134.9 Å^3^. Nitrilotriacetate monooxygenase catalyzes the degradation of the nitrilotriacetate substrate, with the active pocket volume of 5288.4 Å^3^. Ytnj has an active pocket with the volume of 5030.2 Å^3^, but its function remains unknown. Alkane monooxygenase catalyzes the terminal hydroxylation of long-chain *n*-alkanes (C15–C36), and its active pocket volume is 4118.7 Å^3^. The luciferase-like monooxygenase-active pocket volume is 2611.1 Å^3^. The maximum cavity, 8158.3 Å^3^, is that of the EDTA monooxygenase. The figure highlighting the different binding modes of substrate are shown in **Supplementary Figure S4**. We interpreted that the structural variations may be related to the specificities of substrate-binding.

**FIGURE 6:**
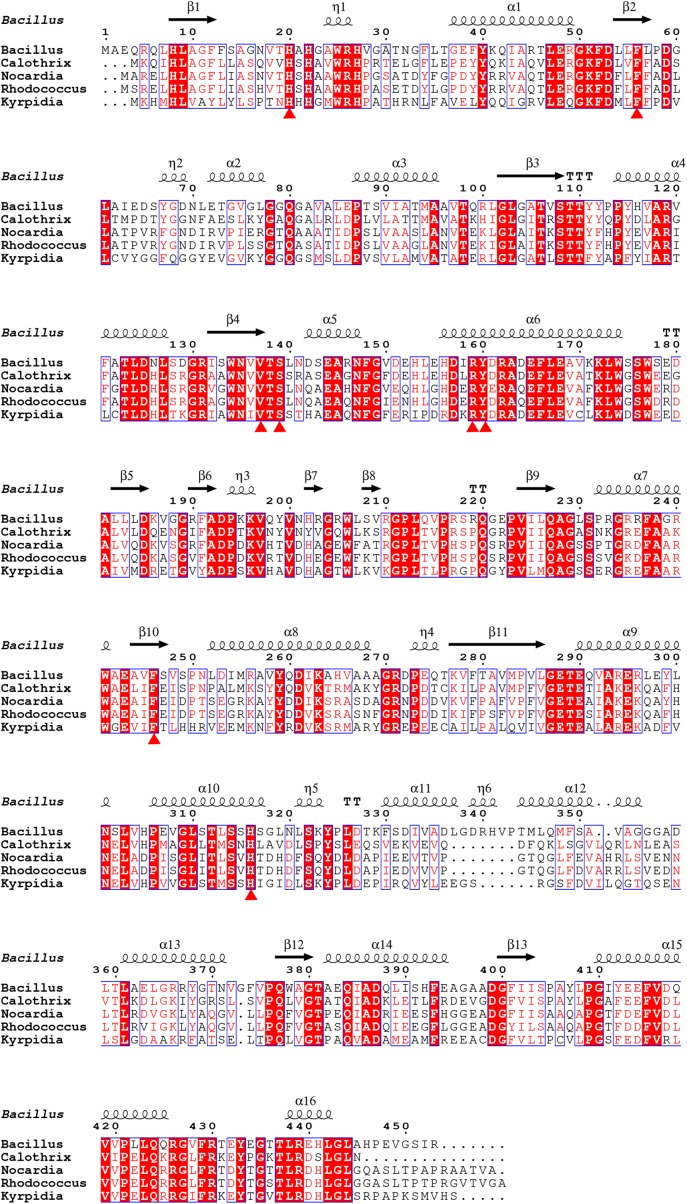
Sequence alignment of BdsA with homologs from different species sharing identities from 48 to 72%. H20, F56, V137, S139, R159, Y160, F246, and H316 are all conserved in BdsA from different species and are denoted by red triangles.

In this study, we have determined the putative residues participating in substrate binding of BdsA through molecular docking and enzyme activity assays. According to the molecular docking result, the substrate-binding site of BdsA may be located on the si-face of the isoalloxazine ring of FMN. As shown in **Figure 3D**, the DBT sulfone is in its stable form and fits well with the active pocket. According to the structure, 13 mutations (six mutations related to FMN-binding and seven mutations related to DBT-sulfone-binding) were constructed. Single mutations in these residues caused near-complete loss in the activity of BdsA. To illustrate the putative substrate-binding site, a sequence conservation analysis was performed, using BdsA homologs from different species with sequence identities from 48 to 72% (**Figure 6**). H20, F56, V137, S139, R159, Y160, F246, and H316 are all conserved in BdsA from different species. As shown in **Figure 3D**, these residues constitute a hydrophobic pocket, which fits well with the substrate DBT sulfone and can stabilize the conformation of the substrate, contributing to the formation of a C4a-(hydro) peroxyflavin intermediate on the si-face of the flavin isoalloxazine ring.

### Mechanism of BdsA

The three-dimensional structure of BdsA is essential for elucidating the catalytic mechanism of the DBT sulfone monooxygenation. Recently, the crystal structure of BdsA was reported by Masahiko at a resolution of 2.8 Å (Okai et al., 2017), which provides architectural information and comparison with the homologous proteins of BdsA. However, the study by Masahiko did not give the structure of the complex with FMN or the substrate. In our study, we determined the crystal structure of BdsA from *Bacillus subtilis* WU-S2B, at a resolution of 2.2 Å, and the structure of the BdsA-FMN complex at 2.4 Å. In addition, we have also constructed a three-dimensional model of the BdsA-FMN-DBT sulfone complex by using flexible molecular docking. These structures may help in the future design of experiments aimed at elucidating the catalytic mechanism in further detail.

**FIGURE 7:**
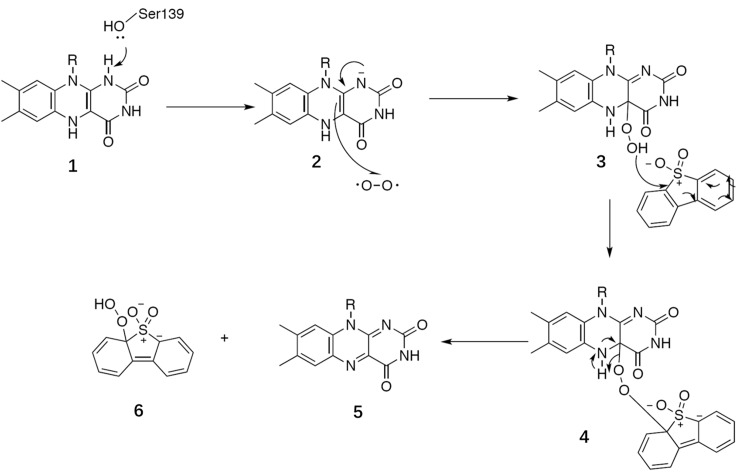
General catalytic mechanism proposed for BdsA.

BdsA is a DBT sulfone monooxygenase that shares a 79% sequence identity with DszA from *Rhodococcus* sp. IGTS8. We reason that the catalysis of BdsA most probably exploits the same mechanism as DszA. Based on the structures we have resolved and previous works on homologous proteins, we propose a general catalytic mechanism for BdsA in sulfinic acid formation (**Figure 7**). The first step is Ser139 as an electron donor accepting the hydride from the N1 atom of the reduced flavin ring to activate FMNH2 (Massey et al., 1969; Bruice, 1984); and the second step of the reaction was a radical combination of 2 (activate FMNH2) to oxygen resulting in flavin hydroperoxide 3, which is the nucleophilicity reagent established in the flavoenzyme-mediated Baeyer–Villiger oxidation of ketones (Walsh and Chen, 1988) and the RutA-catalyzed uracil ring opening reaction (Mukherjee et al., 2010). Then, the addition of compound 3 to DBT sulfone forms the sulfone-stabilized carbanion 4. Succeedent forms 5 (FMN) and hydroperoxide 6.

BdsA play two roles in this process. First, the protein provides an environment that keeps the DBT sulfone and FMN in the correct positions with right orientation (**Figure 3D**). Second, the amino acid Ser139 functions as a nucleophile, activating FMNH_2_. According to the suggested mechanism of DszA (Adak and Begley, 2016), the catalytic cycle is then completed by the conversion of 6 to benzenesulfinic acid and flavin-N5-oxide intermediate to FMN. In the process, the protein only supplies the reaction environment and may not catalyze directly.

## Author Contributions

LG, SX, TS, JS, and SL designed the research. TS, JS, SL, CZ, and JH performed the experiments. TS, JS, SL, YH, SX, and LG analyzed the data and wrote the paper. All authors contributed to the editing of the manuscript.

## Conflict of Interest Statement

The authors declare that the research was conducted in the absence of any commercial or financial relationships that could be construed as a potential conflict of interest.

## Supplementary Material

FIGURE S1 The binding modes of FMN in these homology proteins.

FIGURE S2 The CD spectra result. The wild-type and mutant proteins are shown in different colors.

FIGURE S3 Superposition of the wild and variants amino acids of the active site. The cofactor FMN is shown as yellow sticks. Wild amino acids are shown as cyan sticks, and mutant amino acids are shown as magenta sticks.

FIGURE S4 BdsA, 1yw1, and 3b9o are illustrated in cyan, magentas, and wheat cartoon, respectively.
