# Supplementary figures and images for: Structural and Biochemical Characterization of BdsA from Bacillus subtilis WU-S2B, a Key Enzyme in the “4S” Desulfurization Pathway
Source: Front Microbiol. 2018 Feb 15;9:231. doi: 10.3389/fmicb.2018.00231 (PMC5819316; doi:10.3389/fmicb.2018.00231)

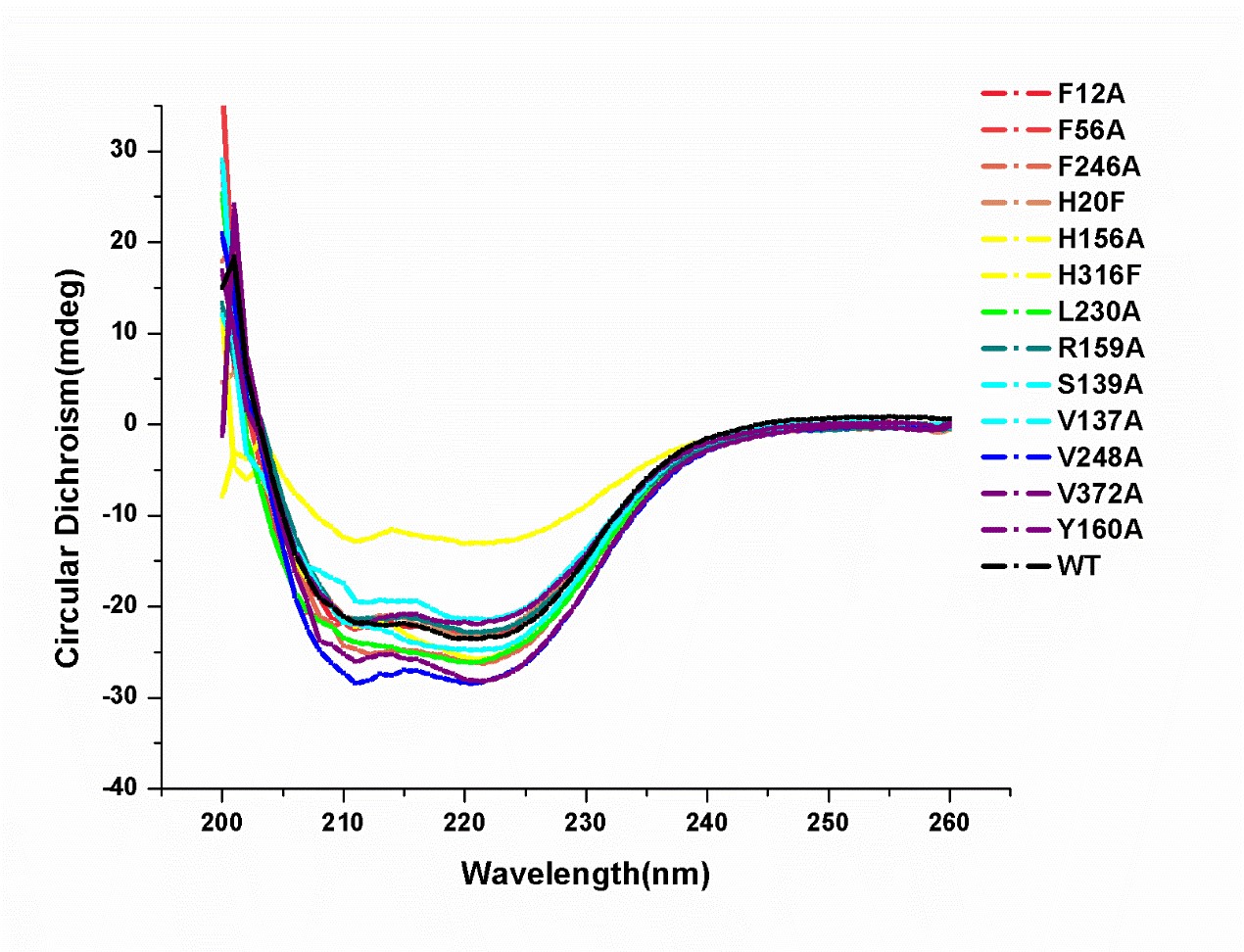

Supplement: FIGURE S1 — The binding modes of FMN in these homology proteins. [file Image_1.jpeg]

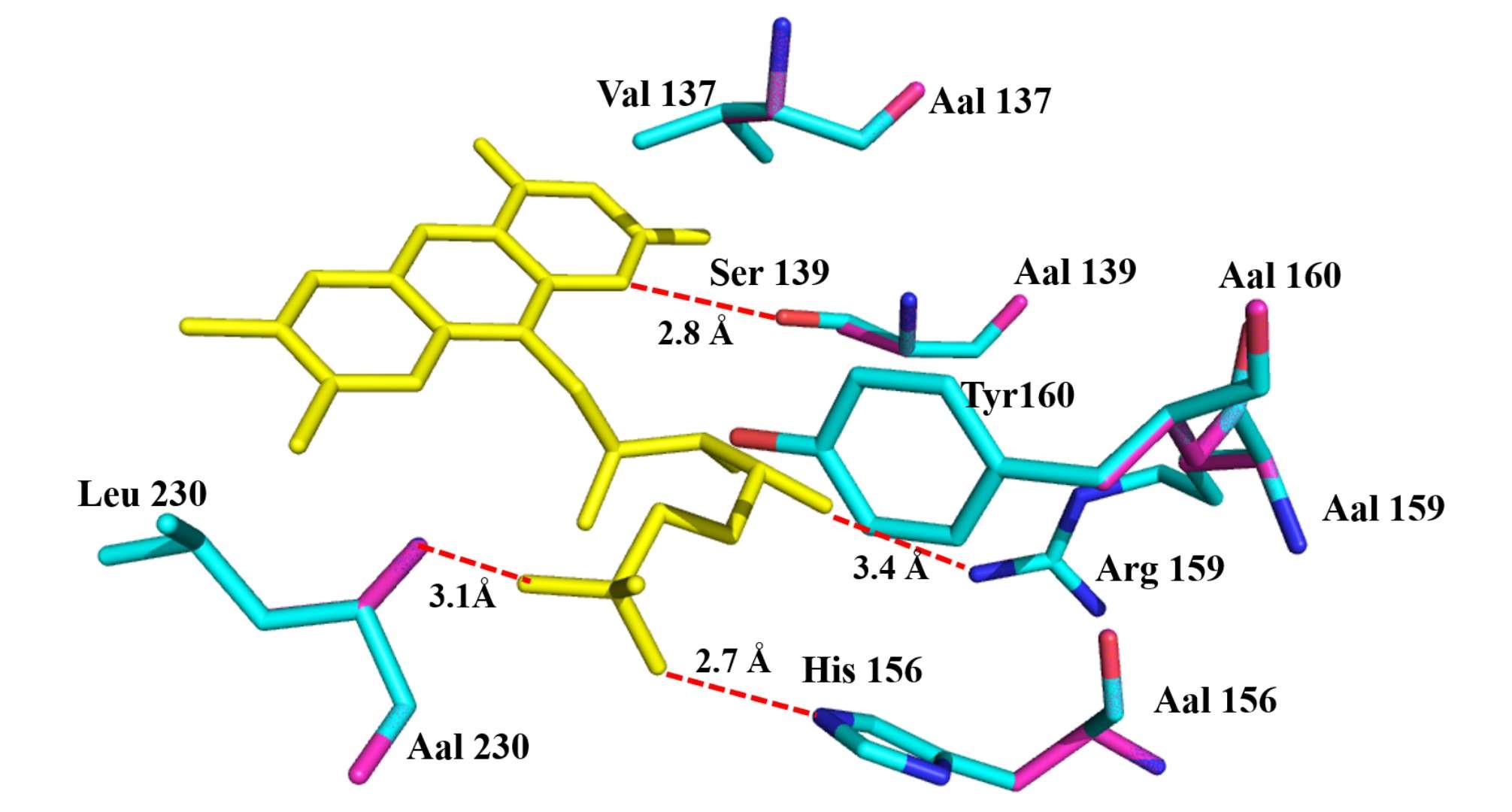

Supplement: FIGURE S2 — The CD spectra result. The wild-type and mutant proteins are shown in different colors. [file Image_2.jpeg]

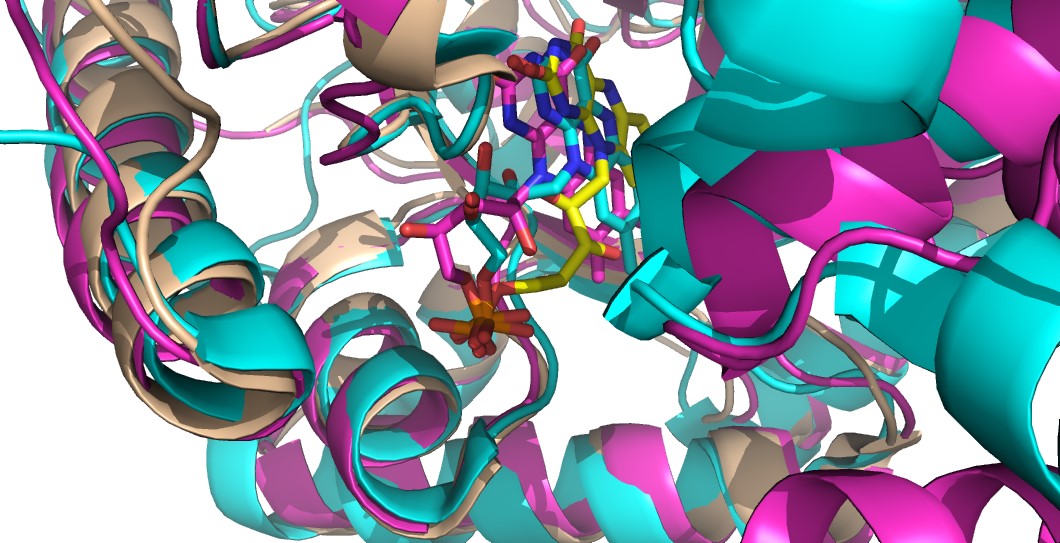

Supplement: FIGURE S3 — Superposition of the wild and variants amino acids of the active site. The cofactor FMN is shown as yellow sticks. Wild amino acids are shown as cyan sticks, and mutant amino acids are shown as magenta sticks. [file Image_3.jpeg]

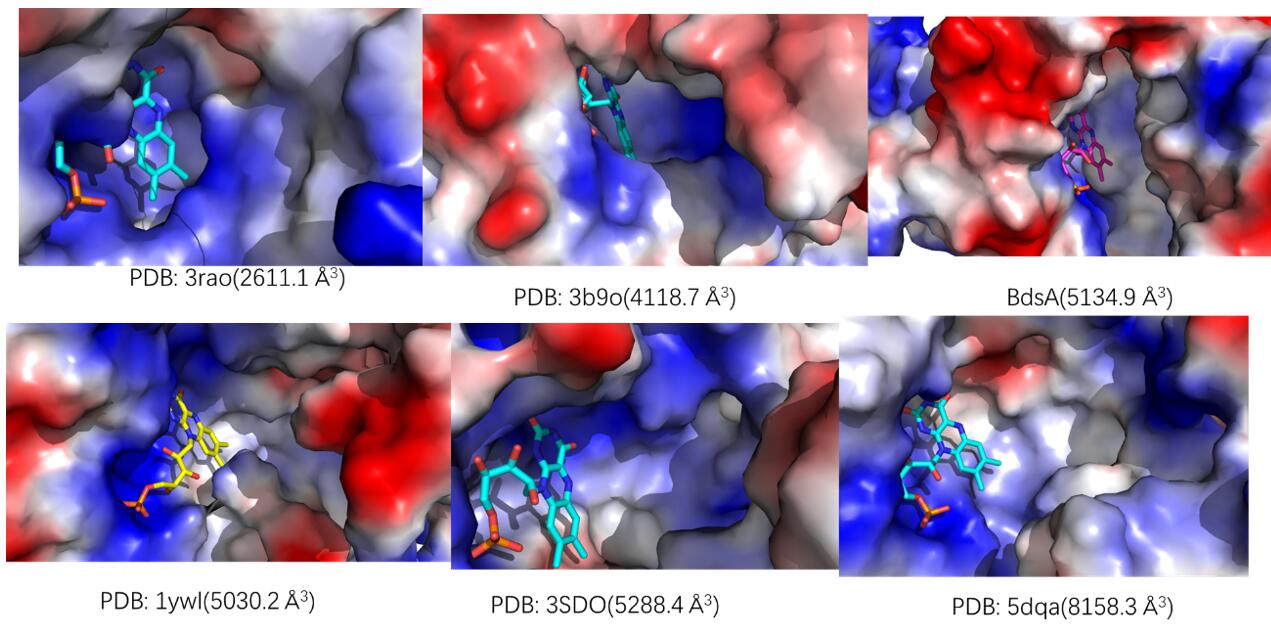

Supplement: FIGURE S4 — BdsA, 1yw1, and 3b9o are illustrated in cyan, magentas, and wheat cartoon, respectively. [file Image_4.jpeg]
